# P-588. Improved Lipid Profile in Virologically Suppressed People with *HIV-1* Switching to Tenofovir DF-Containing, Ainuovirine-Based Compared to Tenofovir Alafenamide-Containing, Boosted Elvitegravir-Based Antiretroviral Regimen: the Secondary Analyses of 48-Week Results of the SPRINT Trial, a Randomized, Active-Controlled Phase 3 Study

**DOI:** 10.1093/ofid/ofae631.786

**Published:** 2025-01-29

**Authors:** Fujie Zhang, Hao Wu, ping ma, Qingxia Zhao, hongxia wei, Hongzhou Lu, Hui Wang, Shenghua He, Zhu Chen, yaokai Chen, Ming wang, Weiping Cai, Hong Qin

**Affiliations:** Beijing Ditan Hospital, Beijing, Beijing, China; Beijing Youan Hospital Affiliated to Capital Medical University, Beijing, Beijing, China; Nankai University Second People's Hospital, School of Medicine, Nankai University, tianjin, Tianjin, China; Zhengzhou Municipal Sixth People’s Hospital and Infectious Disease Hospital of Henan Province, Zhengzhou, Henan, China; The Second Hospital of Nanjing, Nanjing University of Chinese Medicine, Nanjing, Jiangsu, China; Shenzhen Municipal Third Hospital, Shenzhen, Guangdong, China; Shenzhen Municipal Third Hospital, Shenzhen, Guangdong, China; Chengdu Municipal Public Health Clinical Center, Chengdu, Sichuan, China; Chengdu Municipal Public Health Clinical Center, Chengdu, Sichuan, China; Chongqing Public Health Medical Center, chongqing, Chongqing, China; The First Hospital of Changsha, Changsha, Hunan, China; Guangzhou Municipal Eighth People’s Hospital Affiliated to Guangzhou Medical University, Guangzhou, Guangdong, China; Jiangsu Aidea Pharmaceutical Co., Ltd, Yangzhou, Jiangsu, China (People's Republic)

## Abstract

**Background:**

ACC008 is a tenofovir DF (TDF) containing, ainuovirine (ANV), a new-generation NNRTI, based fixed-dose combination (FDC). This FDC has shown noninferior virological efficacy but improved lipid profile compared to tenofovir alafenamide (TAF) containing, boosted elvitegravir (EVG/c) regimen among virologically suppressed people with HIV-1 (PWHs) switching from NNRTI-based regimen. We herein reported the secondary analyses of 48-week lipids profile as prespecified.

**Figure 1.**
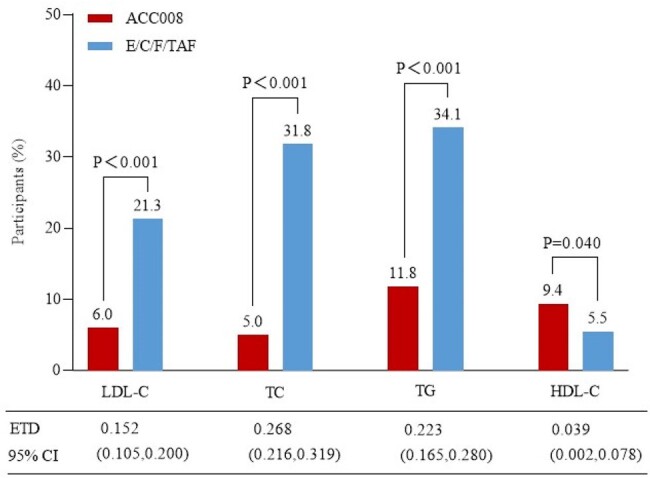
Lipid metabolism adverse events at week 48. ACC008, ainuovirine/ lamivudine/ tenofovir disoproxil; E/C/F/TAF, elvitegravir/ cobicistat/ emtricitabine/ tenofovir alafenamide; HDL-C, high density lipoprotein cholesterol; LDL-C, low density lipoprotein cholesterol; TC, total cholesterol; TG, triglyceride.

**Methods:**

Virologically suppressed PWHs were randomized to ACC008 (n=381) or comparator arm (n=381) for 48 weeks. Changes from baseline (CFBs) in serum lipids were primary safety outcome of interest, including LDL-C, non-HDL-C, total cholesterol (TC), triglycerides (TG), and HDL-C. Other secondary safety outcomes of interest included lipid metabolism adverse events (AEs), and atherosclerotic cardiovascular disease (ASCVD) risk stratification as per the primary prevention target for Chinese low-risk adults.

**Figure 2.**
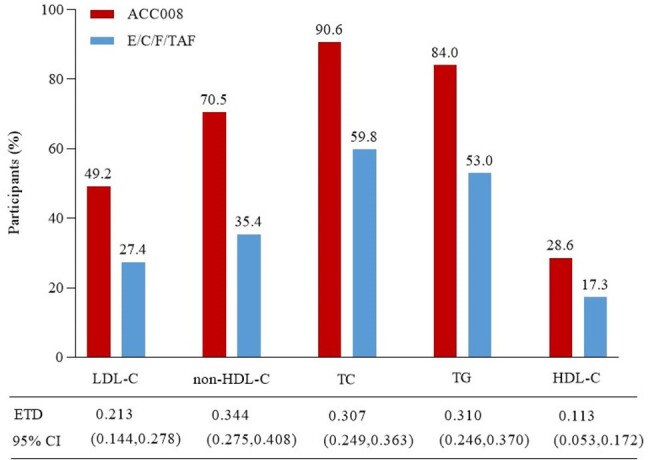
The proportions of PWHs with optimal/acceptable serum lipids and with decreased HDL-C at week 48. ACC008, ainuovirine/ lamivudine/ tenofovir disoproxil; E/C/F/TAF, elvitegravir/ cobicistat/ emtricitabine/ tenofovir alafenamide; HDL-C, high density lipoprotein cholesterol; LDL-C, low density lipoprotein cholesterol; PWHs, peoples with HIV; TC, total cholesterol; TG, triglyceride. For indicators of serum lipids, optimal/acceptable level is defined as LDL-C＜2.6 mmol/L, non-HDL-C ＜3.4 mmol/L, TC ＜5.2 mmol/L, or TG＜1.7 mmol/L, and decreased HDL-C as ＜1.0 mmol/L.

**Results:**

Primary safety outcomes of interest were reported elsewhere. PWHs on ACC008 experienced significantly less frequent lipid metabolism AEs compared to those on comparator (elevated LDL-C, 6.0% vs. 21.3%, estimated treatment difference [95%CI], 0.152 [0.105, 0.200], p< 0.001; elevated cholesterol, 5.0% vs. 31.8%, 0.268 [0.216, 0.319], p< 0.001; elevated TG, 11.8% vs. 34.1%, 0.223 [0.165, 0.280], p< 0.001), but a marginally more frequent decreased HDL-C AE (9.4% vs. 5.5%, 0.039 [0.002, 0.078], p=0.040). The two arms showed a comparable ASCVD risk stratification at the baseline. However, ACC008 arm had a significantly greater proportion of PWHs with optimal/acceptable LDL-C (< 2.6 mmol/L, 49.2% vs. 27.4%, 0.213 [0.144, 0.278], p< 0.01), non-HDL-C (< 3.4 mmol/L, 70.5%, 35.4%, 0.344 [0.275, 0.408], p< 0.001), TC (< 5.2 mmol/L, 90.6% vs. 59.8%, 0.307 [0.249, 0.363], p< 0.001), and TG (< 1.7 mmol/L, 84.0% vs. 53.0%, 0.310 [0.246, 0.370], p< 0.001), but also those with decreased HDL-C (< 1.0 mmol/L, 28.6% vs. 17.3%, 0.113 [0.053, 0.172], p< 0.001) compared to comparator arm at week 48.

**Conclusion:**

TDF-containing, ANV-based ARV regimen improved lipid metabolism in virologically suppressed adult PWHs with noncompromised virologically efficacy compared to TAF-containing, EVG/c-based regimen.

**Disclosures:**

**Hong Qin, MD, PhD**, Jiangsu Aidea Pharmaceutical Co., Ltd: Honoraria

